# Decomposing socioeconomic inequality for binary health outcomes: an improved estimation that does not vary by choice of reference group

**DOI:** 10.1186/1756-0500-3-57

**Published:** 2010-03-04

**Authors:** Vasoontara Yiengprugsawan, Lynette LY Lim, Gordon A Carmichael, Keith BG Dear, Adrian C Sleigh

**Affiliations:** 1 The Australian National University, National Centre for Epidemiology and Population Health, ANU College of Medicine, Biology & Environment, Canberra ACT 0200, Australia

## Abstract

**Background:**

Decomposition of concentration indices yields useful information regarding the relative importance of various determinants of inequitable health outcomes. But the two estimation approaches to decomposition in current use are not suitable for binary outcomes.

**Findings:**

The paper compares three estimation approaches for decomposition of inequality concentration indices: Ordinary Least Squares (OLS), probit, and the Generalized Linear Model (GLM) binomial distribution and identity link. Data are from the Thai Health and Welfare Survey 2003. The OLS estimates do not take into account the binary nature of the outcome and the probit estimates depend on the choice of reference groups, whereas the GLM binomial identity approach has neither of these problems.

**Conclusions:**

The GLM with binomial distribution and identity link allows the inequality decomposition model to hold, and produces valid estimates of determinants that do not vary according to choice of reference groups. This GLM approach is readily available in standard statistical packages.

## Findings

Over the past decade, inequality measures have been adapted from the field of economics and subsequently applied to the study of health inequalities. The concentration index is now widely used to study inequality in the health sector [1-3]. One of its important features is a mathematical property that allows the overall concentration index to be decomposed into a linear combination of concentration indices of its determinants [4,5]. Quantifying contributions of determinants of an overall health inequality has been undertaken for many health outcomes [6-12].

Decomposition estimation was originally designed for cases where the health outcome was continuous using Ordinary Least Squares (OLS) regression. The OLS assumes normality of the outcome variable and implicitly assumes also that the mean outcome is a linear combination of the determinants. Since then another approach, used for the case where the health outcome to be decomposed is binary in nature, has been based on the use of a probit model with marginal effects [2], and Hosseinpoor *et al *[8] modified this approach slightly, using a logit instead of a probit analysis. This extension of the decomposition method to deal with binary outcomes is very appealing, because health sector outcomes are often binary.

Decomposition methodology has long been used to examine discrimination in the labor market [13,14]. The impact of choices of reference groups on parameter estimates for wage discrimination studies was first noted by Jones [15] and then addressed by Oaxaca and Ransom [16] in the context of multiple sets of categorical variables. However, these wage discrimination papers deal with continuous outcomes and provide no information on how to manage reference groups for the binary outcomes often encountered in health studies.

Here we: 1) compare the existing estimation approaches for decomposition of inequality for binary health outcomes; 2) show that the decomposition of a binary outcome using probit analysis can lead to different results with different choices of reference group; and 3) introduce an alternative approach that uses the Generalized Linear Model (GLM) with binomial distribution and identity link.

## Methods

### Data source and variables

We used data from the Thai Health and Welfare Survey of 2003 conducted by the National Statistical Office. In this survey every available member of a sampled household aged 15 years or older was interviewed, a total of 37,202 individuals from 19,952 households.

#### Outcome variable

The health outcome studied was recent morbidity, a binary variable. The English translation of the relevant survey question was: "Have you been ill or not feeling well during the past one month?"

#### Socioeconomic rank

Monthly adult-equivalent household income was used as the measure of socioeconomic status. For Thailand, empirical studies suggest weighting each child aged under 15 as 0.5 of an adult and allowing for economies of scale applying to any household with more than one member by raising adult-equivalent household size to the power of 0.75 [17].

#### Determinants

Three categorical health determinants were examined: eight age-sex groups (males aged 15-29 years, males aged 30-44 years, males aged 45-59 years, males aged 60 years or older, females aged 15-29 years, females aged 30-44 years, females aged 45-59 years, females aged 60 years or older); four levels of education (no education, primary, high school, higher education); and five areas of residence (Bangkok, Central, North, Northeast, South).

Measurement of inequalities in health as a concentration index (C) has primarily drawn on the literature on income inequality measures [3,18,19]. The concentration index can be written in various ways, but one of the most cited is that proposed by Kakwani *et al*. [1]:(1)

Where *h*_*i *_is the variable of interest for the *i*^*th *^person; *μ *is the mean or proportion of *h*;*n *is the number of persons; and if the *n *individuals are ranked according to their socioeconomic status, beginning with the most disadvantaged, then *R*_*i *_is their relative rank, *i *- 0.5/*n*. When there is no inequality (or when inequality is balanced and opposite for equal fractions of the income-ranked population), the concentration index equals 0. If the variable of interest is concentrated at a lower (or higher) socioeconomic level, the concentration index becomes negative (or positive).

Three approaches to the decomposition of a binary health outcome are compared: Ordinary Least Squares (OLS), marginal effects from probit analysis, and Generalized Linear Model (GLM) specifying binomial distribution and identity link [20].

### Ordinary Least Squares (OLS)

Wagstaff *et al *[5] demonstrate that the concentration index of a continuous health outcome can be decomposed into the contributions of individual determinants. In this case, a linear additive relationship between outcome variables *h*_*i *_and the contributions of *k *determinants is appropriate:(2)

and OLS regression is applied to estimate the *β*_*k*_'s. By substituting from Equation 2 into Equation 1, the overall concentration index (C) can be rewritten as a linear combination of the concentration indices of the determinants, plus an error term (Equation 3):(3)

*β*_*k *_are the coefficients from regressions of the health outcome on each *k *determinant,  is the mean or proportion of each *k *determinant, *μ *is the mean or proportion of the health outcome, and *C*_*k *_is the concentration index for the *kth *determinant calculated using Equation 1, replacing the health outcome (*h*_*i*_) with the determinant (x_*ki*_). *GC*_*ε *_is the generalized concentration index for the error term.

### Probit estimates

Health sector variables are seldom continuous and are often binary (e.g., ill, not ill). Van Doorslaer [2] modified Wagstaff's method for use in such non-linear settings. The essential modification was to estimate the *β*_*k*_'s that go into Equation 3 from a probit regression instead of OLS regression. More specifically, van Doorslaer recommends the use of marginal effects of the *β*_*k*_'s. The World Bank technical notes on non-linear estimation suggest generating marginal effects using the Stata command: **dprobit y x**[21]. Marginal effects can also be calculated using the **mfx** command after running the non-linear model. By default, the marginal effects of each explanatory variable are evaluated at sample means, and in large samples the sample mean approximates the overall mean of the marginal effects [22].

### Generalized Linear Models (GLM)

The GLM is an extension of the linear modelling process that allows models to be fitted to data that follow probability distributions other than the normal distribution, such as the binomial distribution [23]. The GLM relaxes the assumption of homogeneity of variances that is usual in linear models and enlarges the class of linear OLS models in two ways:

(i) the distribution of Y for fixed x is assumed to be from an exponential family of distributions [24], which includes important families such as the normal and binomial distributions;

(ii) the relationship between the mean of Y and a linear combination of x's is specified by a link function.

The link function connects the probability distribution of the outcome variable (the random part of the model) to the systematic (explanatory) part of the model. For traditional linear models in which the outcome variable follows the normal distribution, the link function used is the identity link; it specifies that the expected value of the outcome variable is a linear combination of the *x*'s. When the outcome variable follows a binomial distribution, link functions commonly used are the logit and probit, giving rise to logistic and probit regressions respectively.

### Binomial distribution with identity link

The use of GLM with a binomially distributed dependent variable and specifying an identity link function in this non-linear context is a suitable choice in the decomposition analysis of a binary outcome because it considers the structure of the distribution while preserving the link between the independent and dependent variables. The decomposition requires an identity link for the mathematics in Equation 3 to hold. This can be calculated using the Stata command:**glm y x, family(binomial) link(identity)**[25].

## Results

The overall concentration index for reported illness in the previous month in the Thai sample of 2003 was -0.105 (95% confidence interval -0.086, -0.124). Thus recent illness was concentrated more at the poorer end of the income distribution. Proportions in age-sex, education and geographic residence groups are presented in Table 1. Negative concentration indices showed lower socioeconomic status among males aged 60 or older (C = -0.247) females aged 60 or older (C = -0.251), persons with no education (C = -0.321), and those residing in the Northeastern region (C = -0.256). We decomposed the overall inequality observed, estimating contributions due to age-sex, education and regions (Table 2). We used three estimates (OLS, marginal effects from probit analysis and GLM binomial identity) and compared results obtained with two extreme sets of reference groups (the most and least advantaged in each category). Each column presents contributions to the overall concentration index which are obtained from the first element in Equation 3 (i.e., Contribution to Concentration index or CC = ) as well as percentages of the overall concentration index (-0.105). Both the OLS and GLM binomial identity approaches gave CC subtotal estimates that did not vary by choice of reference groups, in marked contrast to the probit-based estimates.

**Table 1:** Proportions and concentration indices for age-sex, education and geographic groups

Groups	Proportion ()	Concentration index (*C*_*k*_)*
**Age-sex (years)**		
Males aged 15-29	0.143	0.035
Males aged 30-44	0.144	0.080
Males aged 45-59	0.103	0.049
Males aged 60+	0.061	-0.247
Females aged 15-29	0.170	0.024
Females aged 30-44	0.176	0.059
Females aged 45-59	0.123	-0.009
Females aged 60+	0.079	-0.251
**Subtotal**	**1.000**	
**Education levels**		
No education	0.055	-0.321
Primary level	0.585	-0.123
High school level	0.258	0.125
Higher level	0.101	0.570
**Subtotal**	**1.000**	
**Regions**		
Bangkok	0.139	0.559
Central region	0.215	0.179
Northern region	0.195	-0.162
Northeastern region	0.341	-0.256
Southern region	0.110	0.024
**Subtotal**	**1.000**	

Source: Thai Health and Welfare Survey 2003

**Table 2:** Contributions to Concentration indices (CC) and its percent contributions (shown in brackets) comparing two reference sets for Ordinary Least Squares (OLS), Probit and Generalized Linear Model (GLM) with binomial distribution and identity link

Groups	OLS (%) Reference 1*	OLS (%) Reference 2*	Probit (%) Reference 1	Probit (%) Reference 2	GLM (%) Reference 1	GLM (%) Reference 2
Males 15-29	ref	-0.007 (7.0)	ref	-0.005 (4.5)	ref	-0.007 (7.0)
Males 30-44	0.002 (-1.7)	-0.015 (14.6)	0.003 (-2.6)	-0.010 (9.3)	0.002 (-1.6)	-0.015 (14.6)
Males 45-59	0.003 (-2.4)	-0.005 (4.8)	0.003 (-3.2)	-0.003 (3.0)	0.002 (-2.3)	-0.005 (4.8)
Males 60+	-0.016 (15.2)	0.006 (-6.1)	-0.020 (18.7)	0.004 (-4.1)	-0.016 (15.1)	0.006 (-6.1)
Females 15-29	0.001 (-0.7)	-0.005 (5.1)	0.001 (-1.0)	-0.004 (3.4)	0.001 (-0.6)	-0.005 (5.1)
Females 30-44	0.005 (-4.7)	-0.010 (9.8)	0.007 (-6.3)	-0.007 (6.5)	0.005 (-4.5)	-0.011 (10.0)
Females 45-59	-0.001 (0.9)	0.001 (-0.7)	-0.001 (1.1)	0.000 (-0.5)	-0.001 (0.9)	0.001 (-0.7)
Females 60+	-0.029 (27.9)	ref	-0.034 (32.2)	ref	-0.029 (27.8)	ref

**Subtotal**	**-0.036 (34.5)**	**-0.036 (34.5)**	**-0.041 (39.0)**	**-0.023 (22.1)**	**-0.037 (34.7)**	**-0.037 (34.7)**

No education	-0.005 (4.6)	ref	-0.005 (5.0)	ref	-0.004 (3.4)	ref
Primary	-0.018 (17.1)	0.002 (-1.6)	-0.019 (18.3)	0.000 (-0.3)	-0.014 (13.2)	0.001 (-0.7)
High school	0.002 (-2.1)	-0.007 (6.3)	0.002 (-2.0)	-0.007 (6.2)	0.001 (-0.8)	-0.006 (5.4)
Higher	ref	-0.016 (15.0)	Ref	-0.015 (14.0)	ref	-0.012 (11.2)

**Subtotal**	**-0.021 (19.7)**	**-0.021 (19.7)**	**-0.022 (21.3)**	**-0.021 (19.9)**	**-0.017 (15.9)**	**-0.017 (15.9)**

Bangkok	ref	-0.039 (37.0)	Ref	-0.034 (32.4)	ref	-0.038 (36.2)
Central	0.001 (-1.3)	-0.018 (17.2)	0.002 (-1.9)	-0.016 (15.1)	0.003 (-2.4)	-0.016 (15.6)
North	-0.016 (15.1)	ref	-0.017 (16.3)	ref	-0.016 (14.8)	ref
Northeast	-0.007 (6.9)	0.037 (-34.7)	-0.008 (8.0)	0.034 (-31.9)	-0.007 (6.2)	0.036 (-34.5)
South	0.001 (-0.6)	-0.001 (0.7)	0.001 (-0.7)	-0.001 (0.6)	0.001 (-0.6)	-0.001 (0.7)

**Subtotal**	**-0.021 (20.2)**	**-0.021 (20.2)**	**-0.023 (21.8)**	**-0.017 (16.1)**	**-0.019 (18.0)**	**-0.019 (18.0)**

**ΣCC**	**-0.078 (74.3)**	**-0.078 (74.3)**	**-0.086 (82.0)**	**-0.061 (58.2)**	**-0.072 (68.6)**	**-0.072 (68.6)**
Residual	-0.027 (25.7)	-0.027 (25.7)	-0.019 (18.0)	-0.044 (41.8)	-0.033 (31.4)	-0.033 (31.4)
**Overall C**	**-0.105**	**-0.105**	**-0.105**	**-0.105**	**-0.105**	**-0.105**

Source: Thai Health and Welfare Survey 2003

*Reference values used in set 1 are: males aged 15-29, higher education, Bangkok.

*Reference values used in set 2 are: females aged 60+, no education, North.

Estimation using marginal effects from probit analysis sees the sum of the contributions to the overall concentration index (ΣCCI) depending on the choice of reference groups, for which no guidance is given in literature that has used this approach. At the two extremes, in our example, choosing set 1 as reference groups tends to result in more of the observed inequality being explained (ΣCC = -0.086, or 82.0 percent of C = -0.105), while choosing set 2 tends to result in appreciably less of it being explained (ΣCC = -0.061, or 58.2 percent of C = -0.105). More generally, it would appear that one is likely to get a higher ΣCC percent figure when reference groups are at the *opposite *extreme to the overall inequality.

## Discussion and conclusion

There are two requirements for a satisfactory concentration index decomposition in a non-linear (binary outcome) setting: *first*, the binomial distribution of the outcome needs to be taken into account; and *second*, the outcome variable must be a linear combination of the independent determinants for the mathematics of the decomposition of the concentration index to hold. The OLS approach is based on a normal model with an identity link function and the probit approach is in essence a binary distribution with a probit link. The OLS approach should not be used for binary outcomes because it does not meet the first requirement. And a probit estimate fails the second requirement because it produces estimates that depend on the choice of reference groups. In practice, it is also possible to estimate a marginal effect using the average of the individual effects rather than the average effect [2].

Decomposition of concentration indices yields useful information regarding the relative importance of various determinants of inequitable health outcomes. But the two decomposition estimation approaches in current use are not suitable for binary outcomes and such outcomes include many useful health indicators. In contrast, our GLM approach specifying the binomial distribution of the outcome and an identity link function allows the decomposition model to hold, and produces valid coefficient estimates that do not vary according to choice of reference groups. In addition the GLM binomial identity link is readily available in standard statistical packages like Stata, and thus should be a valid approach when decomposing concentration indices for binary outcomes.

## List of abbreviations

GLM: Generalized Linear Model; OLS: Ordinary Least Squares; C: Concentration index; CC: Contributions of Concentration indices.

## Competing interests

The authors declare that they have no competing interests.

## Authors' contributions

VY designed the study, analysed the data, and drafted the manuscript. LL was involved in the design of the study and the write-up of the manuscript. GC noticed that probit estimates were sensitive to the choice of reference groups and provided detailed comments and editorial guidance. KD provided conceptual advice. AS guided through to revisions and finalization of the manuscript. All authors read and approved the final version of the manuscript.
